# Tolerance-Inducing Strategies in Islet Transplantation

**DOI:** 10.1155/2012/396524

**Published:** 2012-05-23

**Authors:** Sumantha Bhatt, John J. Fung, Lina Lu, Shiguang Qian

**Affiliations:** ^1^Department of Immunology, Lerner Research Institute, Cleveland Clinic, Cleveland, OH 44195, USA; ^2^Department of General Surgery, Transplant Center, Digestive Disease Institute, Cleveland Clinic, Cleveland, OH 44195, USA

## Abstract

Allogeneic islet transplantation is a promising approach for restoring normoglycemia in type 1 diabetic patients. Current use of immunosuppressive therapies for management of islet transplant recipients can be counterintuitive to islet function and can lead to complications in the long term. The induction of donor-specific tolerance eliminates the dependency on immunosuppression and allows recipients to retain responses to foreign antigens. The mechanisms by which tolerance is achieved involve the deletion of donor-reactive T cells, induction of T-cell anergy, immune deviation, and generation of regulatory T cells. This review will outline the various methods used for inducing donor-specific tolerance in islet transplantation and will highlight the previously unforeseen potential of tissue stromal cells in promoting islet engraftment.

## 1. Introduction

Diabetes mellitus is a disease characterized by metabolic abnormalities and the onset of hyperglycemia, which results from dysregulation of insulin. Insulin promotes the entry of glucose from the blood into the tissues. Diabetes affects approximately 25.8 million people of all ages, with the prevalence rising with age. Long-term complications of diabetes include nephropathy, retinopathy, neuropathy, and atherosclerosis [1]. The type II variant, or noninsulin-dependent form, arises from a resistance to insulin or inadequate production of insulin by the pancreatic *β* islets, eventually leading to islet dysfunction and desensitization to glucose. Type II diabetes accounts for 90–95% of all diagnosed cases of diabetes [1]. The current management for type II diabetes involves a change in lifestyle—weight loss and dietary modifications—and administration of glucose reducing agents. Type I diabetes, the more severe form of diabetes, affects 5% of the population [1] and results from autoimmune destruction of the pancreatic *β* islets. Since the islets fail to produce insulin altogether, the only treatment options for type I diabetes are exogenous delivery of insulin and pancreas or islet transplantation.

Despite improvements in the administration of insulin delivery and insulin supplying devices, maintenance of adequate and steady glucose levels with exogenous insulin therapy alone can be challenging and can cause episodes of hypoglycemia. Diabetic patients with suboptimal control of glycemia ultimately develop long-term complications. Currently the only real “cure” for type I diabetes is transplantation of the pancreas or isolated islets, which would result in insulin production closer to physiological conditions.

However, pancreas transplantation is generally considered only for severe, late-stage diabetics and is a significant surgical procedure requiring extensive immunosuppression [2–4]. Islet transplantation is, therefore, a more feasible alternative to pancreas transplantation. In comparison, islet transplantation has lower risks of morbidity and mortality and greater opportunities for *in vitro* manipulations of islets to optimize engraftment. The concept of transplanting pancreatic fragments to reverse diabetes was first proposed by the English surgeon Watson Williams in 1893 [5]. However, lack of knowledge about immune rejection and immunosuppression at the time prevented progress [2]. It was not until 1967 that Lacy and Ballinger demonstrated the first real advancement in islet transplantation. Using a rodent model in which recipients of islet allografts were induced to develop type I diabetes by administration of streptozotocin (STZ), mimicking autoimmune destruction of the pancreatic islets, Kemp et al. demonstrated that pancreatic islet cell transplants could restore metabolic control and prevent long-term complications [6]. 

Translation from rodent to larger animal and human models had been hampered by difficulties in islet isolation from the pancreas, the lack of quality of isolated islets, and route of administration [7, 8]. The development of an automated method for human islet cell processing by Ricordi et al. in 1986 [9] and the discovery that islet clusters could be heterotopically implanted into the liver contributed to the first successful allogeneic islet transplantation in humans in 1989 by Lacy et al. [6, 10, 11]. Nevertheless, success rates remained low with only 10% of patients achieving insulin independence for greater than a year and demonstrating islet allograft rejection and recurrence of autoimmunity. Poor clinical outcomes were attributed to insufficient numbers of islets being transplanted and an ineffective immunosuppression regimen [12].

## 2. Immune Response to the Graft

The alloresponse is largely a T-cell-mediated response to the major histocompatibility complex molecules (MHCs) on the surface of donor tissues. Antigen presenting cells (APCs), such as dendritic cells (DCs), process and present donor peptides and molecules through MHCs to recipient T cells by the indirect pathway. Additionally, recipient T cells can recognize donor antigen directly on the surface of infiltrating donor-derived APCs through the direct pathway. T-cell receptors (TCR) on the surface of the recipient T cells recognize the peptide-MHC complex, initiating signaling cascades and activation of the T cells. In addition to this primary signal, additional interactions through costimulatory molecules on the T cells and APCs are required for full activation of the T cells. Upon activation, the T cells promote a series of proinflammatory events and initiate the activation of other cell types resulting in recruitment of leukocytes and humoral factors to the graft. The effector response includes the production of the cytokines IFN-*γ* and IL-2 by type 1 helper (Th1) CD4^+^ T cells, the cytotoxic factors granzyme and perforin by CD8^+^ T cells, and alloantibodies (Abs) by B cells [13].

## 3. Pitfalls of Immunosuppression

Current immunosuppressive therapies target T cells since they are the main culprits in rejection. Previously, the standard protocol for immunosuppression for islet transplantation consisted of a combination of calcineurin inhibitors (tacrolimus and cyclosporine), purine analogs (mycophenolate mofetil), and corticosteroids. Many of these agents proved to be diabetogenic, impairing insulin secretion, and lethal to the islets [14]. The advent of the Edmonton protocol in 1999 was a significant step in the field of islet transplantation.

The Edmonton protocol utilized a steroid-free therapy based on low-dose sirolimus, tacrolimus, and daclizumab (a humanized anti-IL-2 receptor *α* mAb). Furthermore, the protocol administered two infusions of islets from different donors to increase chances of engraftment. All 7 patients involved in the trial demonstrated insulin independence beyond 1 year [15, 16]. Despite the success of rapamycin-based therapies, they have their own shortcomings including increased risk of hyperlipidemia, hypertension, and pneumonia [15]. Work by Monti et al. reported that patients conditioned under the Edmonton protocol receiving infusion of cadaveric islets developed lymphopenia and displayed elevated serum levels of homeostatic cytokines, leading to the expansion of autoreactive cells [17].

While the results of multiple center clinical trials demonstrated that the protocol can provide short-term insulin independence and reduce incidences of acute rejection, the patients regressed back to insulin dependency, and graft function was lost in several patients within 5 years [18]. Chronic administration of immunosuppression of any kind will eventually lead to risk of infection, malignancies, and drug toxicity [19]. Maintenance immunosuppression can, therefore, be detrimental rather than beneficial to transplant recipients. Side effects of immunosuppression often outweigh the benefits of islet transplantation.

The risks associated with immunosuppressive agents prompted investigation into tolerance inducing therapies, with the goal being to achieve indefinite graft survival without dependency on long-term immunosuppression while preserving host immunity to other alloantigens. Tolerance induction has been challenging in both large animal and human models due to the complex nature of the alloresponse. Tolerance at both the central and peripheral stages involves clonal deletion of alloreactive T-cells, T cell anergy, immune deviation, and induction of regulatory T cells.

## 4. Central Tolerance

Central tolerance refers to lack of responsiveness to self through deletion of self-reactive T cells in the thymus—the site of T-cell maturation and selection [20]. Donor-specific tolerance can be achieved using strategies similar to those used for preventing autoimmunity. Intrathymic tolerance (IT) can be induced by intrathymic inoculations of recipient APCs pulsed with allopeptides. Alloantigens, when presented in the context of self-APCs result in donor-specific unresponsiveness and promote tolerance. However, the clinical applications of the IT model face many challenges as it is an invasive technique and may have limited potential in adults, since the thymus involutes with age, compromising the tolerance process [21, 22].

An alternative and perhaps more effective method for achieving central tolerance involves the generation of hematopoietic chimerism, which is developed through bone marrow (BM) transplantation. Prospective transplant recipients are conditioned with total body irradiation (TBI) or partial myeloablation prior to BM transplantation in order to make room for the transplanted bone marrow. BM cell transplantation enables the reconstitution of the recipient hematopoietic compartment with donor hematopoietic stem cells. Donor-reactive T cells are then deleted through central regulation and maintained by peripheral mechanisms. The use of TBI for prolonging islet allograft survival was originally proposed by Britt et al. [23] and later shown by other groups to prevent the onset of diabetes in nonobese diabetic (NOD) mice [24]. Though combined islet and hematopoietic cell transplant seems promising, the inherent risks associated with the process have limited its applications. TBI or myeloablation prior to receipt of the BM increases the risk of infection and malignancies. Additionally, BM transplantation is met with the risk of graft versus host disease (GVHD) [25, 26]. Mixed allogeneic chimerism is emerging as a safer method to fully allogeneic chimerism. Mixed chimerism can be achieved in allogeneic BM recipients conditioned with sublethal total body irradiation with the aid of costimulatory blockade or anti-CD4 and anti-CD8 monoclonal (m)Abs [27–29]. NOD mice receiving nonmyeloablative conditioning accepted allogeneic islets and displayed a reversal of autoimmunity, suggesting successful establishment of mixed chimerism [30]. Similarly, NOD mice that received low-dose irradiation, cyclophosphamide (CY), and two intravenous infusions of BM from WT mice showed high levels of donor-cell chimerism and effectively eliminated host donor-reactive lymphocytes after transfer of splenocytes from overtly diabetic NOD mice [31, 32]. BM infusion at the time of islet transplantation may, therefore, be used to induce donor-specific tolerance to islet allografts.

## 5. Tolerance through T-Cell Depletion Strategies

Another strategy in promoting tolerance involves the depletion of alloreactive CD4^+^ and CD8^+^ T cells prior to transplantation. Depletion of alloreactive T cells would promote a hyporesponsive environment and peripheral mechanisms of anergy, thus driving the shift towards tolerance [33, 34]. Depletion can be achieved through TBI, lymphocyte depleting Abs, and pharmaceutical agents. Anti-CD3 mAb has been used for development of mixed chimerism in murine NOD models with few side effects, achieving the same results as myeloablation while bypassing the risk of GVHD [35, 36]. Anti-CD3 mAb is proposed to downmodulate the TCR complex, induce apoptosis of alloreactive T cells, increase production of the immunoregulatory cytokine TGF-*β*, and promote the development of regulatory T cells [37, 38]. Anti-CD3 is effective in inducing tolerance in models of both syngeneic and allogeneic islet transplantation, enabling long-term engraftment [34, 39]. Antithymocyte globulin (ATG) is also a potent inducer of T-cell depletion. Its mechanisms of action are poorly understood, though. Administration of ATG alone or in combination with other agents prolonged allograft survival in various models [40–43]. Islet allograft survival was significantly improved in a nonhuman primate model with administration of ATG and the B-cell-depleting CD20 mAb, rituximab [44]. Taken together these findings suggest that lymphoid-depleting agents may not be effective as a monotherapy but may be useful in combination with other tolerance-inducing therapies.

## 6. Tolerance Induction through Costimulatory Blockade

Tolerance can also be achieved by interfering with costimulatory interactions to inhibit the secondary signal required for full T-cell activation. Suboptimal signaling renders the cells anergic. The B7-CD28 pathway is a key pathway in T-cell activation, survival, and function. Blockade of the B7 (CD80, CD86) receptor on APCs with CD28 on T cells modulates the immune response. In rodent models, B7-CD28 blockade through administration of inhibitory CTLA4-Ig led to prolonged allograft survival and tolerance [45–48]. In nonhuman primates, however, CTLA-Ig treatment alone led to moderate improvements in allograft survival but failed to induce tolerance [47–49].

Similar findings have been reported with CD40-CD154 blockade. Ligation of the CD40 receptor on APCs with CD40 ligand (CD154) on T cells enhances TCR signaling and effector responses [50, 51]. CD154-deficient mice displayed impaired antigen-specific T-cell responses and priming. Coadministration of anti-CD154 mAb and inactivated donor lymphocytes resulted in long-term survival of islet allografts in an STZ-induced rodent model and nonhuman primate models with pancreatectomy [7, 52, 53]. Anti-CD154 mAb downregulated CD28 on donor lymphocytes, thereby inhibiting CD28-CD80 interactions between donor APCs and recipient T cells [54]. Anti-CD154 mAb treatment has been shown to result in “indefinite” survival in islet, BM, and cardiac allograft models [53, 55–57].

Whether true tolerance can be achieved with anti-CD154 mAb alone or in combination with BM transplantation, donor-specific transfusion (DST), or conventional immunosuppression is debatable. Anti-CD154 mAb therapy is met with mixed results. Kenyon et al. reported that rhesus monkeys treated with the humanized anti-CD154 mAb (hu5c8) prior to transplantation with periodic maintenance therapy achieved long-term survival and improved function of intrahepatic islet allografts with little to no side effects [7]. In contrast, Kirk et al., using a similar regimen, found that while hu5c8 therapy prevented acute renal allograft rejection in rhesus monkeys, recipients developed donor-specific Abs and showed signs of chronic allograft nephropathy [58]. A similar result has been reported by Azimzadeh et al. using a primate cardiac allograft model [59]. Although anti-CD154 mAb therapy may allow for the manipulation of donor-specific responses and assist in the induction of tolerance, the consequences of administering anti-CD154 mAb need to be evaluated further. There are strong indications that anti-CD154 mAb therapy results in thromboembolic complications in nonhuman primate models [60]. Discrepancies in outcomes may be due to timing of administration, dosage, duration, and dependent on the animal model.

## 7. DCs in Tolerance

DCs play a critical role in provoking the immune response by mediating the uptake, processing, and presentation of antigen from the microenvironment to naive T cells in the secondary lymphoid tissues. However, DCs also act as regulators in the thymus and periphery by eliminating self-reactive T cells and preventing autoimmunity [21]. Therefore, DCs may be key to achieving central and peripheral tolerance by helping shape the immune response. The immunogenic versus tolerogenic nature of DCs is dependent on the maturation of the DCs and the subset. Manipulation of DCs may, therefore, serve as a therapeutic tool in the design of tolerogenic regimens [61, 62]. Murine DC subsets are characterized and categorized based on their surface markers and origin, such as myeloid DCs (CD11c^+^CD8 *α* ^+^, CD11c^+^CD8 *α* ^−^), plasmacytoid (p)DCs (CD11c^−^B220^+^Gr-1^+^), and Langerhans cell-derived DCs from the epidermis (CD11c^−^). Human DCs fall primarily into two categories: monocytes and pDCs (CD4^+^CD8^−^CD11c^−^). Differences in murine and human DC surface markers make comparisons difficult [63].

Whereas mature myeloid DCs upregulate MHC class II and the costimulatory molecules CD40, CD80, and CD86, immature DCs downregulate these markers and are potent inhibitors of allospecific T-cell responses [64]. The lack of stimulatory molecules allows immature DCs to induce antigen specific hyporesponsiveness in T cells [65]. Immature DCs also vary in their stimulatory activity. CD11b^+^CD8 *α* ^−^ DCs induce a Th2 phenotype (IL-4, IL-5, IL-10, and IL-13), while CD11b^−^CD8 *α* ^+^ DCs induce a Th1 phenotype (IL-2 and IFN-*γ*) through secretion of IL-12 [63, 66]. In addition to skewing the Th1/Th2 profile, tolerogenic DCs have been shown to promote allograft acceptance by deletion and anergy of alloreactive T cells and induction of donor-specific regulatory T (Treg) cells [67–69].

The ability of tolerogenic DCs to mediate anergy and proliferative arrest of alloreactive T cells has been demonstrated by Munn et al. pDCs produce indoleamine 2,3-dioxygenase (IDO), which catabolizes the essential amino acid L-tryptophan, and triggers the integrated stress pathway within antigen specific T cells and suppresses their proliferation and function [70]. The exact mechanism by which these tolerogenic DCs inhibit alloreactive T cells is not well understood but is thought to involve the activation of the general control nondepressible-2 (GCN2) kinase pathway. The GCN2 pathway is activated in response to an accumulation of uncharged tRNAs that results from amino acid deprivation [71]. Transfer of tolerogenic IDO producing immature DCs from primary tolerant recipients into murine cardiac allograft recipients enhanced allogeneic T cell apoptosis and Treg-cell development resulting in prolonged graft survival [67]. Ochando et al. showed that treatment with DST and anti-CD154 mAb prompted pDCs to migrate to the allograft and subsequently induced Treg development in the lymph nodes. Furthermore, pDCs isolated from tolerogenic mice promoted CD4^+^CD25^−^Foxp3^−^ T cells to convert into CD4^+^CD25^+^Foxp3^+^ Treg cells *in vitro* [72].

DC maturation can be limited through addition of cytokines (IL-10 and TGF-*β*) and costimulatory blockade [73, 74]. In mice, BM cells cultured in the presence of granulocyte macrophage colony-stimulating factor (GM-CSF) alone tended to acquire an immature phenotype—expressing low levels of MHC class II and costimulatory molecules. In contrast, addition of IL-4 to the GM-CSF culture led to DC maturation and high levels of MHC class II, CD40, CD80, and CD86 expression [75–77]. DCs can be restricted to an immature state through pharmacological interventions as well. *In vitro*, rapamycin conditioned BM-derived DCs suppressed the development of alloreactive CD4^+^ T cells but allowed for the proliferation and functioning of Tregs [78]. Lutz et al. examined the tolerogenic potential of immature myeloid DCs and found that the transfer of these immature myeloid DCs induced a state of T-cell unresponsiveness and resulted in a significant improvement in cardiac allograft survival [75].

In addition to extrinsic influences, DC maturation is also governed by NF-*κ*b signaling. NF-*κ*b regulates expression of MHCs and costimulatory molecules. Lu et al. inhibited NF-*κ*b activity within DCs by constructing a decoy double-stranded oligodeoxynucleotide (ODN) that selectively inhibited expression of costimulatory molecules while permitting the expression of MHCs, thus preventing DC maturation. Genetic engineering of DCs allows for expression of molecules that play a role in the inhibition of T-cell responses. Administration of DCs deficient in NF-*κ*b activity prevented the onset of diabetes in NOD mice [79]. Recipients of virally transduced DCs expressing CTLA4-Ig, IL-10, or FasL displayed improved pancreatic islet and cardiac allograft survival [80–83]. The therapeutic potential of these engineered DCs is limited by the fact that the cells may become activated or mature *in vivo* through exposure to the virus and proinflammatory stimuli [80].

## 8. Myeloid-Derived Suppressor Cells (MDSCs) in Tolerance

MDSCs are emerging as important regulators of tolerance. Originally identified for their role as suppressors in cancer [84, 85], MDSCs are comprised of heterogeneous myeloid cell populations: subsets of DCs, macrophages, and granulocytes. MDSCs in mice are characterized based on expression of the markers CD11b, Gr-1, Ly6C, and Ly6G. MDSCs can be divided even further by their nuclear morphology as mononuclear, monocytic MDSCs (CD11b^+^Gr-1^high^Ly6G^−^Ly6C^high^) and polymorphonuclear, granulocytic MDSCs (CD11b^+^Gr-1^int^Ly6G^+^Ly6C^low^) [86]. Their variability can even be extended to their function and production of immunosuppressive components arginase-1 (Arg-1), inducible nitric oxide synthase (iNOS), and reactive oxygen species (ROS), with suppressive function depending on the environment and pathological condition [87–89]. MDSCs make up a relatively small proportion in healthy mice but expand and accumulate in the lymphoid tissue of tumor bearing mice, remaining in an immature state and inhibiting antitumor responses [90, 91]. The generation and expansion of MDSCs is dependent on inflammatory cues [92, 93]. The inflammatory molecules vascular endothelial growth factor (VEGF) and GM-CSF have been linked to the accumulation of MDSCs [94, 95]. Additional proinflammatory cytokines IL-1*β* and IL-6 have been shown to contribute to the development of MDSCs. IL-1*β* secreting tumors had greater accumulation of MDSCs with improved lifespan resulting in aggressive tumor growth [96–98].

The ability of MDSCs to suppress T-cell responses and expand Treg cells has been demonstrated in various models of autoimmunity, infection, and cancer [99–101]. The mechanisms by which MDSCs impart their suppressive effect include production of iNOS and arginase-1. Arginase-1 depletes L-arginine resulting in downregulation of the TCR*ζ* chain and inhibition in production of cell cycle regulator proteins [102, 103]. iNOS promotes phosphorylation of the Janus kinase 3 and STAT5 pathway and inhibits MHC class II expression and T-cell proliferation [104–106]. MDSCs also produce ROS and peroxynitrates, which inhibit protein tyrosine phosphorylation through nitration of tyrosine residues [88, 107]. Increased production of these factors was observed in tumor models and related to T-cell unresponsiveness [108].

As suggested in tumor models, MDSCs may be useful in transplant settings by suppressing alloreactive T-cell responses and prolonging graft survival. In a murine kidney allograft model, Dugast et al. demonstrated that recipients with long-term surviving grafts exhibited an accumulation of CD3^−^MHC II^−^CD11b^+^CD80/86^+^ cells in the blood and graft. Isolation and characterization of the cells revealed myeloid-like morphology. These cells showed strong suppressive activity against activated T cells, with suppressive activity related to the increased number of cells and dependent on production of iNOS. The phenotypic and functional analysis of these cells fits the criteria for MDSCs. Inhibition of the MDSC suppressor molecule iNOS by aminoguanidine resulted in rejection of kidney allografts [109]. MDSCs also have implications in skin transplant models. Heme oxygenase-1 (HO-1) secreting MDSCs facilitated tolerance in recipients of skin allografts through T-cell suppression and IL-10 production [110]. Our group has recently shown that cotransplantation of MDSCs with islet allografts reduces CD8^+^ effector T-cell responses and results in the expansion of antigen specific Tregs in the draining lymph nodes (dLNs), spleen, and peripheral blood, translating to markedly improved islet allograft survival. MDSC-mediated suppressor functions were dependent on the inhibitory B7-H1 (PD-L1)-PD1 pathway. The protective effect imparted by Tregs was negated in recipients receiving B7-H1^−/−^ MDSCs. Frequencies of CD4^+^Foxp3^+^ cells were markedly reduced in all compartments in B7-H1^−/−^ MDSC recipients. Direct evidence for the role of B7-H1 in MDSC-induced Treg development was demonstrated through *in vitro* coculture of donor BALB/C T cells with DCs or MDSCs from WT or B7-H1^−/−^ MDSCs. WT MDSCs expanded Foxp3^+^ cells, whereas MDSCs deficient in B7-H1 exhibited reduced capacity for Treg induction. Further evidence was shown in B6 mice intravenously injected with OVA-specific CD4^+^ T cells with subsequent footpad injection of OVA-pulsed WT or B7-H1^−/−^ MDSCs. Examination of the popliteal LNs showed an increase in the frequency of CD4^+^Foxp3^+^ cells in recipients of OVA-pulsed WT MDSCs compared to recipients of OVA-pulsed B7-H1^−/−^ MDSCs. Therefore, it appears that MDSCs require B7-H1 to exert their immunoregulatory activity and to induce Tregs [111, 112].

## 9. Tregs in Tolerance

The presence of CD4^+^CD25^+^Foxp3^+^ regulatory T cells is correlated with improved graft outcomes and tolerance [113–116]. Tregs act as surveillance for the immune system, and depletion of Tregs results in lymphoproliferation and autoimmune syndrome [117–119]. Biopsies taken from grafts of tolerant recipients had greater infiltration of Tregs compared to those from rejecting grafts [120]. While Treg cells only make up 5–10% of the mature T-cell population, they are potent in numbers [121]. Lymphodepletional therapies and costimulatory blockades alone can do little in terms of promoting tolerance; therefore, strategies that promote Treg cells and their functions will improve chances of engraftment. Unfortunately, many immunosuppressive agents, especially those targeting the IL-2 receptor and IL-2, inhibit Treg development and function since IL-2 signaling is critical to T cell survival and proliferation [122]. The ideal scenario would minimize immunosuppressive therapy and focus on expanding the endogenous Treg population or generation of antigen-specific Tregs, thereby inducing tolerance without the need for immunosuppression.

There is evidence that apoptotic cells have the ability to influence Treg development by way of DCs [123]. Apoptotic cells suppress inflammatory responses by emitting inhibitory signals to DCs and other phagocytes. Adopt transfer of donor apoptotic cells imparted an immature phenotype on DCs, which in turn inhibited CD4^+^ T cell activation and IL-2 and IFN-*γ* production. Combination with CD40-CD154 blockade led to prolonged cardiac allograft survival through induction of Tregs [124].

It is speculated that Tregs impart their suppressive function through direct interactions with cells (engagement with CTLA4-Ig), production of soluble factors and inhibitory cytokines TGF-*β* and IL-10, and cytokine deprivation [125, 126]. Zhang et al. [127] provided mechanistic insight into how Tregs exert their suppressive effect using an islet allograft model. Tregs in the blood migrated to the allograft through the guidance of the chemokines CCR2, CCR4, and CCR5. Upon activation, they traveled to the dLNs where they inhibited DC migration and antigen-specific T-cell migration and response in the dLNs and islet allografts [127]. Similar observations were made by Golshayan et al. Alloantigen specific Tregs were expanded *in vitro *and maintained their suppressive function *in vivo*. When transferred into recipients of skin allografts, they trafficked to the secondary lymphoid organs and accumulated in the graft dLNs and within the allograft itself. The donor-specific Tregs delayed graft rejection in the absence of immunosuppression. Tregs infiltrated skin allografts early on in the immune response and suppressed rejection by inhibiting alloreactive T-cell responses. In the presence of Tregs, CD4^+^ T cells produced far less IFN-**γ** and did not accumulate in the secondary lymphoid tissues [113]. The suppressive features of Tregs make them candidates for therapeutic use in islet transplantation.

The differential effects of immunosuppressive therapies on Treg development make it difficult to determine the optimal combination of agents that promote Treg activity, while inhibiting Teff functions. Transient depletion of dividing T cells with anti-CD25 mAb altered the homeostatic balance and created space for *de novo* expansion of Tregs. Anti- CD25 mAb recipients displayed tolerance to islet allografts, unlike control-treated recipients [128]. These data suggest that not all T-cell depleting therapies may have an effect on Tregs or that Tregs may adapt by downregulating CD25. In contrast, Li et al. showed that anti-CD25 mAb treatment prevented tolerance of liver allografts by reducing the ratio of CD4^+^CD25^+^ Tregs to CD3^+^ T cells [129]. The Teff/Treg ratio determines the outcome of the graft; therefore, expansion of Treg populations and deletion of effector T-cell populations are crucial to tolerance induction. Zheng et al. have shown that combined treatment of rapamycin and agonistic IL-2/Fc and antagonistic mutant IL-15/Fc selectively targeted alloreactive T cells while preserving Tregs [130]. When administered with other calcineurin inhibitors, beneficial effects were lost [122, 131, 132]. Thus, not all agents within a family necessarily exert the same effect.

One of the current limitations of Treg therapy is the inability to generate sufficient numbers of antigen-specific Tregs for therapeutic outcomes. The absence of reliable markers for human Tregs makes isolation and purification difficult. Isolated T cells would have to be expanded *in vitro*, but expansion may not be restricted to Treg populations specifically. Additionally, Tregs demonstrate a great degree of plasticity and have the potential of converting to an effector phenotype *in vivo* [133]. The risks associated with Treg therapy warrant further investigation and require technical advancement before application in humans.

## 10. Organ Stromal Cells in Tolerance

The contribution of organ stromal cells in the regulation of the immune response is understudied. Our group has extensively investigated the influence of these populations in the liver on islet transplantation. The liver is unique in that it is one of few organs with inherent tolerogenic properties [134–136]. A number of factors have been attributed to the tolerogenic state of liver allografts including increased B-cell infiltration, production of soluble MHC class I antigens, involvement from stromal cells, and presence of Tregs [112, 137–139]. The importance of liver stromal cells is highlighted by the fact that while liver transplantation in mice results in indefinite acceptance, transplantation of hepatocytes is rejected [140, 141].

We have focused on a population of stromal cells in the liver called the hepatic stellate cells (HSCs), which have been known to have a primary role in liver fibrosis and repair following hepatic injury. Additional features of these cells are their participation in the storage of vitamin A (retinoid) droplets, and their ability to produce TGF-*β* in response to inflammation [142–144]. However, little is known about the involvement of HSCs in immune regulation. HSCs, from both mice and humans, have been shown to act as nonprofessional APCs and upregulate MHCs, CD40 and CD80, and inhibitory PD-L1, in response to proinflammatory cytokines [137, 139]. Jiang et al. found that HSCs can also expand CD4^+^CD25^+^Foxp3^+^ cells in an IL-2-dependent manner [145]. Therefore, it is conceivable that these cells possess tolerogenic qualities. Addition of HSCs into an MLR culture at an HSC to T ratio of 1 : 20 resulted in 80–90% inhibition of T-cell response [137]. *In vivo* inhibition was demonstrated in mice by cotransplantation of HSCs with islet allografts. A prolongation in survival in >60% of islet allografts was observed without immunosuppression. This was associated with elimination of antigen-specific T cells and enhanced MDSC and Treg development [112, 146, 147]. It appears that HSCs exert their effects primarily by inducing MDSCs. The ability of HSCs to promote MDSCs was verified *in vivo* by analyses of mononucleocytes infiltrating the islet grafts. Cotransplantation with HSCs led to an accumulation of MDSCs, instead of DCs, as seen in islet alone grafts. *In vitro* evidence confirmed that addition of HSCs to BM-derived DC culture promoted the development of CD11b^+^CD11^−^c cells displaying suppressive functions. MDSC induction was abolished in IFN-*γ* ^−/−^ recipients, demonstrating the additional dependency of an inflammatory environment for MDSC development [112, 146]. MDSCs have been shown to induce Treg development as well [148]. Increased Treg levels were observed with islet/HSC transplantation. We, therefore, suspect that HSCs recruit MDSCs to the islet allografts and promote engraftment upon inflammation. Benten et al. have shown the tolerogenic role of HSCs in promoting hepatic engraftment in a rat hepatic allograft model. Hepatic transplantation led to the activation of HSCs and a series of genetic and phenotypic changes within the HSCs. Prior depletion of HSCs impaired hepatocyte acceptance [149]. HSCs demonstrate potent immunomodulatory properties and can influence the development of suppressor cells. With further study, HSCs may be implemented into tolerance inducing therapies.

Analogous to HSCs, Sertoli cells (SCs) within the seminiferous tubules of the testis also exhibit suppressive features. The immunoprivileged SCs have been exploited for protection of various transplanted tissues [150–154]. The immunoprotective capabilities of SCs can be extended across allogeneic and xenogeneic barriers [155, 156]. Transplantation of SC xenografts alone into NOD mice altered the cytokine milieu in the pancreas and induced a regulatory environment, inhibiting IL-6, IL-10, and IFN-*γ* production while promoting TGF-*β* and the regulatory enzyme indoleamine. TGF-*β* produced by the SCs was responsible for the generation of autoantigen-specific regulatory T cells. Recipients displayed reversion of diabetes with the SC xenografts [157]. It has also been shown that SCs have the ability to influence and inhibit T-cell responses and complement activation, although the mechanisms are not well defined [158–160]. Selawry et al. were the first group to demonstrate the application of the immunomodulatory SCs for inducing tolerance in islet transplantation [158, 161, 162]. Cotransplantation of SCs and islet allografts induced long-term graft survival, with recipients remaining normoglycemic for at least 60 days after transplantation without systemic immunosuppression [150, 157]. The transfer of xenogenic neonatal porcine SCs (NPSCs) at the time of islet transplantation was shown to prolong islet allograft survival in nonimmunosuppressed rats in a dose-dependent manner (MST = 16.33 ± 1.53 days versus islets alone group, 5.67 ± 0.94 days). Examination of grafts showed reduced lymphocyte infiltration and increased expression of Bcl-2 compared to recipients receiving islets alone, suggesting that NPSCs may also be regulating the expression of immunoprotective genes [163]. There is also evidence that NPSCs can suppress the upregulation of CD40 expression on DCs in response to LPS stimulation, thereby preventing full activation of the DCs and inducing the development of tolerogenic DCs [160]. The efficacy of NPSCs has even been demonstrated in human models of xenotransplantation. Transplantation of porcine islets and NPSCs into type 1 diabetic patients led to a reduction in insulin dependence and maintenance of metabolic control for up to 4 years without immunosuppression in half of the 12 patients involved in the study [164]. Cotransfer of SCs at the time of transplantation may, therefore, provide protection for alloislet transplants and improve chances of engraftment.

The BM also contains a rich source of immunomodulatory stromal cells referred to as mesenchymal stem cells (MSCs). MSCs have the capacity to develop into various types of mesodermal tissues and exhibit properties of self-renewal [165]. MSCs alter the cytokine profile of DCs, naive and effector T cells, and NK cells in response to IFN-*γ*, downregulating the production of IFN-*γ* and TNF-*α* in the microenvironment and inducing a more tolerogenic phenotype. IFN-*γ*-induced expression of the immunosuppressive factors TGF-*β*, hepatic growth factor (HGF), IL-10, prostaglandin E2, matrix metalloproteinases (MMPs), and IDO account for the inhibition of alloresponses [166–168]. Furthermore, MSCs were found to promote the expansion of Tregs [169–171]. Characterization of human MSCs has been a challenge, and the results are somewhat conflicting [165, 172]. Fiorina et al. found that in their NOD model, murine bone-marrow-derived MSCs were positive for the stem cell markers CD29, CD44, CD73, CD105, and CD166 and negative for hematopoietic lineage markers CD45 and CD90.2 and costimulatory molecules CD40, CD80, and CD86. Interestingly, the MSCs expressed high levels of PD-L1, and it was speculated that the level of PD-L1 expression enabled the immunosuppressive functions of MSCs [169].

The ability of MSCs to modulate T-cell responses and influence tissue rejection suggests a therapeutic role for inducing tolerance in islet transplantation. The negligible expression of MHC II and absence of costimulatory molecules on the cell surface allow MSCs to escape immune recognition. However, the immunomodulatory effects of MSCs observed *in vitro* have been difficult to replicate *in vivo*, as evidenced in baboon skin allograft and rat cardiac allograft models [173, 174]. Allogeneic MSC infusion has been effective in preventing the onset of diabetes in prediabetic mice and reversing hyperglycemia in diabetic mice [169, 175]. In islet transplantation models, MSCs demonstrate the ability to migrate to the pancreatic islets and exert an immunosuppressive effect in the graft microenvironment [176, 177]. MSCs have also been shown to influence pancreatic vascularization and remodeling following transplantation [178]. Although the mechanisms by which MSCs exert their immunosuppressive effects remain elusive, Ding et al. suggest that one possible strategy involves the production of MMP-2 and MMP-9. In culture, MSCs mediated T-cell proliferative arrest and hyporesponsiveness by downregulating CD25 on the surface of T cells. Downregulation of CD25 was dependent on the production of MMP-2 and MMP-9 by MSCs, since inhibition of MMP via the thiirane gelatinase inhibitor SB-3CT restored surface expression of CD25 and T-cell responses. Administration of syngeneic MSCs at the time of syngeneic islet transplantation prevented rejection in murine STZ-induced diabetic Rag-2/*γ*(c)KO recipients reconstituted with CD4^+^CD25^−^ T cells and resulted in a rapid return to normoglycemia. In contrast, recipients under the same conditions treated with the MMP-2 and MMP-9 inhibitor SB-3CT became diabetic within 30 days (MST = 30 days) [167]. In theory, MSC therapies sound promising in the induction of tolerance but require further evaluation before clinical application.

## 11. Concluding Remarks

The future of islet transplantation depends on the development of tolerance inducing therapies. While temporary immunosuppression can be advantageous, the long-term risks outweigh the benefit. Tolerance suggests freedom from insulin dependency and an improvement in the patient's overall quality of life. A tolerizing regimen that utilizes techniques that selectively target donor-reactive T cells while expanding populations of regulatory T cells will result in better outcomes. Further investigation into inherently tolerogenic cells in the body such as HSCs, SCs, and MSCs will aid in the design of therapies. Though many challenges still remain, the progress made in the animal models of tolerance holds great promise for humans.
